# Do Sleep-Related Metacognitive Strategies Shape My Sleep? The Relationships between Strategies for Controlling Sleep-Related Intrusive Thoughts and Subjective and Objective Sleep Quality in Young Adulthood and Older Age

**DOI:** 10.3390/brainsci13020271

**Published:** 2023-02-06

**Authors:** Enrico Sella, Elena Carbone, Erika Borella

**Affiliations:** Department of General Psychology, University of Padova, 35131 Padova, Italy

**Keywords:** sleep quality, actigraphy, metacognition, thought control strategies

## Abstract

This study examined the associations between thought control strategies and subjective and objective sleep quality, across the adult lifespan. One hundred forty-nine individuals without insomnia (age range 18–86 years; M = 45.35, SD = 20.53) completed the Thought Control Questionnaire Insomnia–Revised for assessing sleep-related thought control strategies. Self-reported sleep quality was measured with the Pittsburgh Sleep Quality Index. Then, subjective and objective sleep parameters (i.e., total sleep time, sleep onset latency, sleep efficiency) were recorded through a sleep diary and an actigraph across 7 days. Results from linear mixed-effects models showed that a worry strategy was associated with longer subjective sleep latency and shorter subjective total sleeping time. An aggressive suppression strategy was associated with longer subjective total sleeping time. No such involvement of thought control strategies was detected for subjective sleep efficiency and all of the objective sleep parameters. Other individual differences (i.e., age, sex, circadian preference, self-reported sleep quality) also explained both subjective and objective sleep parameters, though to a different extent depending on the sleep parameter considered. The assessment of sleep-related thought control strategies, along with other individual characteristics, should be considered to account for individual differences in sleep quality and implement practices/interventions to support it in adulthood and older age.

## 1. Introduction

Sleep represents a fundamental function that allows one’s body and mind to restore. It plays a crucial role in promoting physical and mental health and supporting quality of life throughout the entire life span [1]. Several changes in sleeping patterns and sleep quality occur in adulthood, especially in older age [2,3], and the consequences can negatively affect health and daily functioning. With aging, people report changes in their sleep patterns and circadian rhythms, often starting in middle age and increasing as people grow older [4,5]. In particular, older adults tend to go to bed earlier, have more difficulty falling asleep, wake up frequently during the night, spend more time awake at night, and have low sleep efficiency [2,3,4], with consequences for their quality of life (see [1], for a review). 

Various factors can either counteract or trigger as well as maintain sleep difficulties over time. Among these factors there is sleep-related metacognitive activity, such as certain strategies for controlling intrusive and unwanted thoughts before sleep [6,7,8,9]. Such sleep-related metacognitive strategies, also called thought control strategies, encompass worry (focusing on other problems), aggressive suppression (critically analyzing and judging one’s thoughts), social avoidance (preventing thoughts with the emotional support of someone such as a bed partner or refusing to discuss them), reappraisal (reinterpreting one’s thoughts), cognitive distraction (shifting attention to other thoughts), and behavioral distraction (doing something physically). Some of these strategies are assumed to be adaptive strategies to contrast sleep difficulties and promote sleep quality. For example, distraction efforts (i.e., cognitive or behavioral distraction strategies) involve recognizing that a stressor exists and represent the attempt to focus away from it, in the short term at least (e.g., [10]). However, other strategies more likely lead to the initiation, progression, and maintenance of sleep difficulties and disorders, such as insomnia. Worry, aggressive suppression, social avoidance, and reappraisal were found to be associated with poor sleep quality in people with insomnia [6,11,12], and thus, they are considered maladaptive strategies to manage sleeping difficulties and attempts to fall asleep [6,7]. Having one’s mind “race out of control” just before sleep, as presented by theoretical models of insomnia [7] and in findings from several studies [13,14], can affect one’s sleep quality in terms of difficulty in sleep initiation and/or maintenance [7,15,16]. Individuals with insomnia are known to adopt maladaptive thought control strategies frequently, in attempting to fall asleep and trying to solve their sleep difficulties on a typical night [6,7]. 

The few studies exploring the association between such thought control strategies and sleep quality in young and older adults without insomnia also found worry, aggressive suppression, and reappraisal related to self-reported poor sleep quality [8,17,18]. Worry and reappraisal strategies were then shown to play a major role in predicting self-reported poor sleep quality when a broad age range sample (from 18 to 79 years old) was involved [9]. However, better sleep quality was found to be associated with cognitive distraction in young adults [17] and behavioral distraction in older adults [8], corroborating the notion that such thought control strategies might more likely be adaptive to manage sleep difficulties in people without sleeping disorders.

To note, the few studies addressing the associations between thought control strategies and sleep quality in people without insomnia focused on overall self-reported sleep quality—defined as feeling refreshed in the morning and generally satisfied with one’s sleep. However, sleep is a multifaceted health construct [19], comprising different sleep parameters that contribute to sleep quality indicators throughout lifespan [3]. Among such parameters, total sleep time (TST; defined as the time spent asleep during the night), sleep onset latency (SOL; defined as the total time needed to fall asleep), and sleep efficiency (SE; defined as the total time asleep out of the total time in bed) are typically evaluated when using sleep diaries and/or actigraphy methods in aging studies (e.g., [20]), and during the course of insomnia treatment (see [21]), thus representing key variables of interest in sleep quality. Only one study to date examined the associations between sleep parameters assessed with an objective sleep measure (via actigraphy) and thought control strategies in a sample of older adults [8]. Sella et al. [8] found that only the worry strategy was associated with longer objective time needed to fall asleep (i.e., SOL), while none of the other strategies was related to objective sleep parameters (i.e., TST and SE). This issue is worth investigating using both subjective and objective parameters of sleep quality, because older adults commonly report and experience numerous changes in sleeping patterns and quality [2,3] that might not be captured considering overall sleep quality. 

In light of the gathered evidence, the present study aimed at further elucidating the relationship between sleep-related metacognitive strategies (i.e., thought control strategies) and objective and subjective sleep quality in a sample of individuals spanning a broad age range, with a typical aging process and without sleeping disorders. Towards this aim, we considered here for the first time different subjective and objective, classically used sleep parameters (i.e., TST, SOL, and SE), which were recorded across 7 consecutive days with a sleep diary and an actigraph (see also [8]). Longitudinal data collection on sleep–wake patterns—with both subjective and objective measures—though rarely used in adulthood and older age (e.g., [8,19]), is indeed recommended.

Considering the (very sparse) evidence assessing thought control strategies and (overall) self-reported sleep quality in adults without sleeping disorders [8,9,17,18], we could hypothesize reappraisal and worry strategies, along with aggressive suppression, to be associated with worse subjective sleep parameters (as recorded here), thus having a dysfunctional role in subjective sleep quality. Previous reports showed weak or null associations between the other thought control strategies (e.g., cognitive and behavioral distraction, social avoidance) and (overall) self-reported sleep quality in adults with a typical aging process and no insomnia [8,9]; thus, we expected the same for the subjective sleep parameters considered here.

Concerning objective sleep parameters, because sleep-related metacognitive strategies are usually adopted before sleep [6], and in the light of previous evidence collected on older adults without insomnia [8], we particularly expected to find an association between the worry strategy and longer objective SOL, as well as weak or null associations between all thought control strategies and other objective sleep parameters (i.e., TST and SE).

Nonetheless, because all of the sleep parameters of interest were examined here with a 7-day collection of recordings and in a sample with a broader age range, we explored whether a different pattern of associations between thought control strategies and subjective and objective sleep parameters might emerge.

Finally, given that age, sex, circadian preference (i.e., morningness), and self-reported sleep quality account for differences in subjective sleep parameters ( e.g., age: [3], sex: [22], circadian preference: [23,24], self-reported sleep quality: [8,9]) and—albeit modestly and less consistently—objective sleep parameters (e.g., age: [9], sex: [22], circadian preference: [19,25,26,27], self-reported sleep quality: [8,9]), these variables were controlled.

## 2. Materials and Methods

### 2.1. Participants 

The study involved 149 adults between 18 and 86 years of age (M = 45.35; SD = 20.53; 90 females, 59 males; see Table 1). All participants were community dwellers and were recruited by word of mouth. 

All participants were healthy, and they met the following inclusion criteria: (a) no depression, with scores under the clinical cut-off of 10 on the Geriatric Depression Scale for adults over 64 years old [28] and under 14 on Beck’s Depression Inventory II for middle-aged and young adults (BDI-2; [29]); (b) no excessive symptoms of anxiety, as measured by the State-Trait Anxiety Inventory Y2 (STAI-Y2; [30]); that is, more than 2 SDs from the mean in the Italian normative data [31]; (c) no signs of cognitive impairment or incipient dementia (for people older than 64 years) when tested with the short version of the Italian Checklist for Multidimensional Assessment adopted in the Veneto region to assess older people, taking a score of 9 out of 10 as a cut-off (SVAMA; [32]). None of the participants reported a history of head trauma, psychiatric, neurological, physical diseases, or sleep disorders (e.g., insomnia), as assessed via semi-structured interviews [33]. Participants also completed the Pittsburgh Sleep Quality Index (PSQI; [34]) to assess self-reported sleep quality (considered as covariate in the models, see below), and the Morningness–Eveningness Questionnaire–Reduced Version (MEQ-r; [35,36]) to assess circadian preference (considered as covariate in the models, see below).

The study protocol was carried out following the recommendations of the local university’s research ethics committee (No. 2049) and in accordance with the Declaration of Helsinki [37].

### 2.2. Materials 

#### 2.2.1. Measures of Subjective and Objective Sleep Quality

Subjective sleep parameters: Sleep diary. Participants used sleep diaries to record sleeping and waking patterns and provide information about sleep-related behavior for 7 days. Every morning and evening for a week, participants completed an adapted version of the sleep diary from the National Sleep Foundation Diary (see [8]). The following three subjective sleep parameters (dependent variables) were considered in the study: TST (i.e., minutes recorded as sleep during the night); SOL (i.e., minutes elapsed between lights off and the first sleep period); and percentage of SE (i.e., the ratio of TST to time spent in bed).

Objective sleep parameters: Actigraphy. The Actiwatch-64 (AW-64; Phillips Respironics) is a small movement-detecting device used to record participants’ sleeping and waking states over 7 days. Actigraphic data were recorded in 1 min epochs with a medium threshold setting and were analyzed using Actiware 6.2 software (Phillips Respironics, [38]). In this study, three objective sleep parameters (dependent variables) were examined: TST (i.e., the number of minutes scored as sleep between lights off and lights on); SOL (i.e., the number of minutes between lights out and the first period scored as sleep); and SE (i.e., the ratio of TST to total time in bed (lights off to lights on).

#### 2.2.2. Sleep-Related Metacognitive Strategies

The TCQI-R [6] is a 35-item questionnaire that measures how frequently a person uses specific metacognitive strategies while being kept awake by thoughts on a 4-point Likert scale ranging from 0 (never) to 4 (almost always). Five thought control strategies were investigated: aggressive suppression (Cronbach’s α = 0.74), cognitive distraction (Cronbach’s α = 0.64), behavioral distraction (Cronbach’s α = 0.77), worry (Cronbach’s α = 0.73), and reappraisal (Cronbach’s α = 0.62). The social avoidance subscale was not considered in the analyses because its internal consistency was not acceptable (Cronbach’s α = 0.34; which was also found by [9,17]). The dependent variables were the five strategies, where higher scores indicated stronger use of metacognitive strategies.

### 2.3. Procedure

The participants completed ad hoc semi-structured interviews to assess the presence of sleep disorders, and then they completed the following questionnaires: the SVAMA, the PSQI, the MEQ-r, the BDI-2 (young adults) or the GDS (older adults), and the STAI-Y2. Then, participants also completed the TCQI-r, filled out a sleep diary, and wore an actigraph for the next 7 consecutive days in a naturalistic setting (the participants’ homes).

### 2.4. Statistical Analyses

Linear mixed effects (LMEs) model analyses were conducted to investigate the relationship between subjective and objective sleep parameters measured across 7 days (i.e., TST, SOL, and SE), demographics (age and sex), circadian preference (MEQ-r), self-reported sleep quality (PSQI), and all of the thought control strategies. All of the LMEs models were run using the lme4 package (version 1.1–31; [39]) in R software [40]. Considering the multilevel nature of the daily measures (subjective and objective sleep parameters) and simultaneously accounting for both among- and within-subject sources of variation [41], subject was included as a random factor for all of the models. To find the relevant predictors for each objective and subjective sleep parameter, a model comparison approach was used, and predictors of interest were subsequently included into more parsimonious models, starting from a null model (intercept only) to a full model (i.e., including all predictors). Therefore, we first ran the null model (m1) [(′y ~ intercept + (random1|participantNumber)]. Then, a model including demographics, circadian preference, and self-reported sleep quality (m2) [′y ~ intercept + age + sex + MEQ-r + PSQI + (random1|participantNumber)] was computed. Finally, in the full model, all of the thought control strategies (m3) [′y ~ intercept + age + sex + MEQ-r + PSQI + TCQI-r, Agg Supp + TCQI-r, Cogn Dist + TCQI-r, Behav Dist + TCQI-r, Reapp + TCQI-r, Worry (random1|participantNumber)] were added. Each model was compared to the previous one using the Akaike information criterion (AIC; [42,43,44]). The most plausible model for each objective and subjective sleep parameter was the one with the lowest AIC value and the larger AIC weight (following [42,43]; MuMIn package, [45]). As we included nine predictors in each tested model (see below), a posterior power analysis was computed with G*Power [46], and our sample size (149 participants) allowed us to detect a medium effect size (f^2^ = 0.20) with a significance of 0.05 and a power of 0.98 for multiple linear regression.

## 3. Results

Table 1 shows the descriptive statistics for participants’ demographic characteristics, circadian preference, self-reported sleep quality, subjective and objective sleep parameters, and all of the thought control strategies. 

### 3.1. Subjective Sleep Parameters 

Table S1 (Supplementary Material) shows the coefficients of the best model for each subjective sleep parameter (as measured by sleep diary) across the week (for further details, see Supplementary Material). 

#### 3.1.1. Total Sleep Time

**Figure 1:**
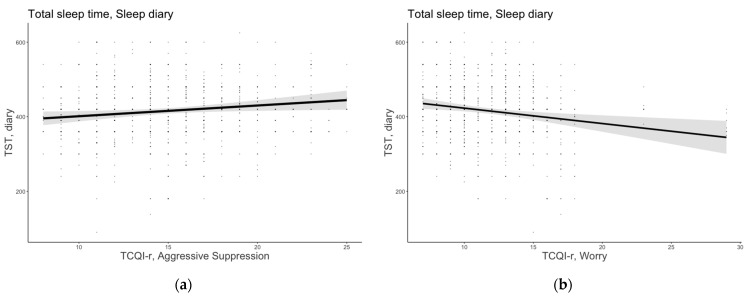
Subjective total sleep time as a function of (**a**) aggressive suppression and (**b**) worry strategies. The error bars represent 95% confidence intervals of the estimated means.

The model that included age, sex, MEQ-r, PSQI, and the thought control strategies emerged as the most plausible for TST (AICw = 0.999; conditional R^2^ = 0.385). Aggressive suppression was a positive predictor of TST (B = 2.88, 95% confidence interval [CI] [0.45, 5.30]), suggesting that individuals who adopted such a metacognitive strategy reported spending more time asleep. The worry strategy emerged as a significant and negative predictor of TST (B = −4.13, 95% CI [−6.66, −1.59]), indicating that those who employed such a dysfunctional strategy reported having shorter sleep duration. Figure 1 shows the significant predictor effects of aggressive suppression and worry strategies on subjective TST. 

Moreover, an effect of age and PSQI emerged for TST (B = −0.84, 95% CI [−1.26, −0.41], B = −6.12, 95% CI [−10.69, −1.54], respectively): older people and people who reported having sleeping difficulties reported having lower sleep duration. Sex and circadian preference, as well as the other thought control strategies, did not significantly contribute to explaining TST.

#### 3.1.2. Sleep Onset Latency

**Figure 2:**
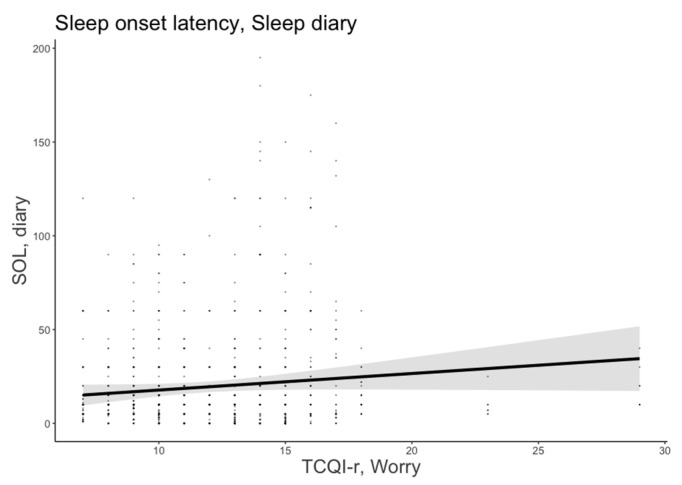
Subjective sleep onset latency as a function of worry strategy. The error bars represent 95% confidence intervals of the estimated means.

The most plausible model for SOL included age, sex, MEQ-r, and thought control strategies (AICw = 0.84; conditional R^2^ = 0.478). The worry strategy was a significant predictor of subjective SOL (B = 1.57, 95% CI = [0.54, 2.60]), indicating that people who focused on worries before sleeping spent more time trying to fall asleep. Figure 2 shows the significant predictor effects of worry strategy on subjective SOL.

In addition, age and PSQI were significant and positive predictors of SOL (B = 1.56, 95% CI [0.56, 2.57], B = 2.48, 95% CI [0.66, 4.30], respectively). Older age and overall self-reported sleeping difficulties were significantly associated with a much longer time to fall asleep. No other significant predictors emerged.

#### 3.1.3. Sleep Efficiency

The model that included age, sex, MEQ-r, and PSQI showed better plausibility (AICw = 0.991; conditional R^2^ = 0.441). Lower PSQI scores (B = −1.48, 95% CI [−2.17, −0.78]), indicating fewer self-reported sleeping difficulties, were associated with better ratios of time spent asleep to time spent in bed. Older age was a significant predictor of SE (B = −0.13, 95% CI [−0.19, −0.07]), indicating that older individuals reported lower SE. Sex and MEQ-r did not contribute significantly to explaining SE.

### 3.2. Objective (Actigraphic) Sleep Parameters

Table S2 (Supplementary Material) reports the most plausible models concerning objective sleep parameters (for further details, see Supplementary Material).

#### 3.2.1. Total Sleep Time

The full model was selected as the best model (AICw = 0.93; conditional R^2^ = 0.329), but none of the considered variables showed plausibility to predict objective TST. 

#### 3.2.2. Sleep Onset Latency

The model including age, sex, MEQ-r, and PSQI showed better plausibility for objective SOL (AICw = 0.95; conditional R^2^ = 0.410). Age (B = 0.07, 95% CI [−0.03, 0.17]), sex (B = −4.22, 95% CI [−8.08, −0.37]), and PSQI (B = 1.26, 95% CI [0.10, 2.42]) were significant predictors of objective SOL, indicating that older age, being male, and having self-reported sleeping difficulties led to longer time to fall asleep. MEQ-r did not contribute significantly to explaining SOL.

#### 3.2.3. Sleep Efficiency 

The model with age, sex, and circadian preference (MEQ-r) emerged as the best model in predicting objective SE (AICw = 0.66; conditional R^2^ = 0.804). A lower MEQ-r score (indicating evening preferences; B = −1.23, 95% CI [−2.11, −0.35]) was a significant predictor of better objective SE. No other significant variables emerged.

## 4. Discussion

This study examined the associations between sleep-related metacognitive strategies (i.e., thought control strategies) for controlling intrusive and unwanted thoughts at bedtime and different subjective and objective sleep parameters. In particular, for the first time, we explored whether and to what extent different thought control strategies could affect differently specific subjective and objective sleep parameters (i.e., TST, SOL, and SE) measured over 7 consecutive days in adults and typically aging individuals without sleep disorders.

The results from our regression models allowed us to clarify the contribution of thought control strategies to subjective and objective sleep quality parameters.

Regarding subjective sleep parameters, partially in line with the previous literature [8,9,17,18] and our expectations, our results newly show that specific thought control strategies were associated with subjective sleep quality parameters. Their roles also changed depending on the sleep parameter considered. 

In particular, the worry strategy predicted both TST and SOL parameters: individuals who frequently used the worry strategy in their efforts to control or manage intrusive and unwanted thoughts at bedtime seem more likely to report shorter sleep duration (i.e., shorter TST) and to spend much more time attempting to fall asleep (i.e., longer SOL). In line with our expectations and consistent with studies involving clinical (e.g., [6,12,47]) and nonclinical samples [9,47,48,49], our findings highlight that focusing on excessive worries at bedtime worsens sleep duration and latency, decreasing individuals’ sleep duration and making it more difficult for them to fall asleep, and this appeared also true throughout adulthood and into older age for individuals presenting with no insomnia.

Of interest, our results revealed that the use of aggressive suppression was associated with longer TST. This finding appeared in contrast with evidence showing that individuals with insomnia [6,11,12], or self-reported poor sleepers compared to good sleepers [8,9], reportedly use such a strategy. Suppression efforts imply the recall of negative and harmful thoughts people want to suppress (e.g., [50]), thus producing a well-known paradoxical rebound effect in a variety of psychological disorders (e.g., [51]), including insomnia disorders. However, when adopting such a strategy in the absence of insomnia, as here, aggressive suppression appears to have an adaptive and/or functional role in individuals with no sleep disorders: participants’ self-regulatory emotional and cognitive resources still function at bedtime, thereby favoring the reduction of intrusive and unwanted thoughts before sleep and improving, in turn, the amount of time spent sleeping. It will thus be interesting in future studies to examine and better understand the role and impact of sleep-related metacognitive strategies on sleep quality depending on the presence, or not, of psychological and/or sleep disorders. 

None of the other thought control strategies (e.g., cognitive and behavioral distraction, reappraisal) were involved in explaining subjective SOL and TST. These null findings seem to suggest that specific self-regulatory metacognitive/behavioral mechanisms, grounded in some of the thought control strategies, affect sleep aspects such as the time needed to fall asleep and the overall sleeping time. Such an issue also merits further examination.

However, no such involvement of thought control strategies was detectable for subjective SE. The efforts to control intrusive and unwanted thoughts at bedtime appeared to have a limited influence on the ability of individuals without sleep disorders to sleep at night. SE is more sensitive to sleep schedule [52,53] and behavioral practices, not only at bedtime (which includes the decision to get in bed), but also upon waking in the morning (which includes the decision to get out of bed), thereby determining SE [53]. Therefore, the use of thought control strategies at bedtime might be limited to the early part of the night (because the time typically spent struggling to fall asleep was spent sleeping) or to the time spent asleep (because of the total time asleep after the time spent trying to fall asleep), rather than the perceived ratio between actual sleep duration and length of time spent in bed (which can cover a larger time period).

Regarding objectively assessed sleep, none of the thought control strategies were related with the objective sleep parameters recorded here. Such a pattern of results is contrary to previous findings [8] and our expectations. It is worth mentioning, however, that we recorded objective sleep parameters over 7 days and in a sample spanning a broad range of age, which could account for these contrasting findings. 

Overall, such a differential effect of thought control strategies on subjective and objective sleep parameters further corroborates the notion of a discrepancy between subjective and objective sleep parameters, as found in the sleep literature [54,55] and found here (see Supplementary Material, Table S5). Various underlying mechanisms seem to be associated with either subjective or objective sleep parameters (e.g., greater inter-individual night variability; [56]); among others, the thought control strategies used to approach sleep appear to be related more with subjective than objective sleep patterns in individuals without insomnia (at least). 

Our models also allowed the role of other individual characteristics (i.e., age, sex, circadian preference, and self-reported sleep quality) to emerge in explaining subjective and objective sleep parameters, here again with a different pattern of results depending on the sleep parameter considered. 

In line with the literature and our expectations, age predicted worse subjective sleep parameters: older age was found to be associated with greater sleep latency and decreased sleep duration and efficiency. When objectively measured parameters are considered, although age predicted increased objective sleep latency (consistent with previous evidence [2,3]), it did not predict sleep duration and objective sleep efficiency. In other words, older age per se is not associated with objective poor sleep quality, but other health conditions would seem to play a greater role (see [1,57], for reviews). 

As for sex, no significant influence on subjective or objective sleep parameters emerged, except for objectively measured sleep latency: being male was associated with greater difficulties falling asleep. This result could be explained because males in our sample had a higher mean age than females had (i.e., the average age for male participants was 46.49, SD = 22.7, and the average age for female participants was 44.60, SD = 19.07); this age difference was also more pronounced if we considered participants 60 and older (male participants in this group were on average 74.30 years old, SD = 7.12, whereas female participants had an average age of 68.92, SD = 5.33), a pattern in line with [57]. 

As for circadian preference, it did not prove to be significantly involved in explaining subjective sleep parameters. This could be because circadian preference is mainly associated with sleep timing/habits (i.e., bedtime and waketime), e.g., [23,24,25,26,27], rather than the perception of sleeping quality per se. Interestingly, in fact, circadian preference was significantly related with objective SE, with a better sleep efficiency that was found to be associated with eveningness (i.e., people who prefer evening activities) but not with TST or SOL. This finding might relate to individual differences in sleep schedule during the week. It is in fact plausible that evening people tend to spend less time sleeping during weekdays because their social and work commitments (for those working) force them to wake up earlier with respect to their preferred sleeping times, causing them to sleep poorly [27] and thereby accumulate a “sleep deficit”. Consequently, evening people have to sleep longer and recover their sleep deficit by increasing their actual/objective SE during the weekend [27]. This sleep schedule might instead not be sufficient to modify or influence subjective sleep parameters or other specific objective sleep parameters (e.g., TST and SOL), which would appear to be more sensitive to factors related more to psychological components (e.g., dysfunctional/maladaptive sleep-related metacognitive activity as measured here) than they are to behavioral ones (e.g., adopting inconsistent sleep behaviors/habits). Future studies should confirm this speculation. 

Finally, in line with previous evidence [8,54,55], poor self-reported sleep quality (i.e., higher PSQI scores) significantly predicted all of the subjective sleep parameters considered here (i.e., shorter TST, longer SOL, and lower SE), whereas it was only associated with longer objective SOL. This latter result suggests that overall self-reported sleeping quality also affects the objective time needed to fall asleep [8]. 

The lack of associations between PSQI and other objective parameters (TST and SE), which contrasts with the associations found among the subjective parameters, highlights again the inconsistency between subjective and objective sleep measures, at least in individuals without insomnia [54,55]. Such a result could also be explained by our participants’ reduced—though experienced/reported—sleeping difficulties. Some individuals had scores above the PSQI cut-off of 5 [34]. Although none exceeded a PSQI score of 7 out of 21 (see Table 1), i.e., none suffered from sleep disorders, there might be individuals experiencing some sleep “difficulties” that do affect their perceived sleep quality more than their actual sleep (potentially leading to worsened sleeping difficulties in the long run).

Some limitations of this study deserve to be acknowledged. First, other characteristics relating to sleep-related metacognitive activity (e.g., beliefs about sleep; [8,58]), pre-sleep cognitive and somatic arousal (e.g., worries), and negative or positive emotions (e.g., [59]), not considered here, would help shed light on the role of thought control strategies at bedtime and sleep quality. In addition, because physiological arousal has been found to predict sleep disturbance [60,61], the perseverance of using a maladaptive strategy such as the worry strategy at bedtime can also be accompanied by physiological activation that can explain difficulty falling asleep (as reported above) and staying asleep throughout the night. Since sleep quality is also defined by other sleep indicators/parameters (e.g., nocturnal awakenings, nap frequency; [3]), future studies to better capture the nature of strategies should thus make the effort to consider a wider number of sleep quality indicators. The use of polysomnography to more accurately examining objective sleep parameters is also recommended. Although actigraphy is a noninvasive and valid method to measure objective sleep–wake parameters and motor-based activity in the participant’s home, it has some limits such as overestimating sleep quantity, as shown in certain clinical samples (people with sleep disorders or other clinical disorders) [62]. Future directions might then include studies that examine, alongside subjective sleep measures, objective sleep measures using polysomnography for assessing other micro- and macro-structural sleep parameters (e.g., waking after sleep onset, or sleep fragmentation index) and other psychological metrics (e.g., skin conductance or heart rate variability) for a more in-depth assessment of psychological and psychophysiological indices of using a sleep-related metacognitive strategy at bedtime.

In conclusion, our results highlight that thought control strategies more likely influence subjective than objective sleep quality. Furthermore, they suggest how the role of certain thought control strategies varies depending on the subjective sleep parameter considered, thereby offering some insight and recommendations for future clinical and applied perspectives. For instance, the use of worry strategies for controlling and managing intrusive and unwanted thoughts at bedtime was associated with individuals’ increased time falling asleep and decreased subjective sleep duration. Thus, reducing people’s involvement with their mental activity before bed (also defined as “racing mind”; [7]), rather than targeting intrusive and unwanted thoughts in general or changing content-related thoughts related to sleeping difficulties (as done by individuals typically adopting worry strategies; [6]), might be a recommended strategy to change the way in which they approach sleep. Suppression efforts to control intrusive thoughts at bedtime could also be suggested as an adaptive/functional thought control strategy, at least in terms of perceived longer sleep duration in people without sleep disorders. Targeting people’s self-regulatory resources when they experience intrusive and unwanted thoughts at bedtime and guiding them to use suppression in a functional way (at bedtime), along with other sleep hygiene practices (e.g., practicing relaxing activities at bedtime such as meditation, creating a comfortable sleep room), could be a recommended approach to support their sleep quality.

Overall, our results suggest the importance of (a) including the assessment of sleep-related thought control strategies, (b) considering individual characteristics to identify individuals who are more prone to report poor sleep quality in the long run, and then (c) tailoring sleep interventions (e.g., nonpharmacological sleep hygiene programs) to teach methods for managing sleep-related metacognitive strategies in order to contrast negative changes in sleeping patterns and quality throughout adulthood.

## Figures and Tables

**Table 1:** Mean (M) and standard deviations (SD) of the sample’s demographic characteristics and the measures of interest.

	M	SD
Age (years)	45.35	20.53
Female (%)	60%	-
Education (years)	14.95	3.96
Circadian preference		
MEQ-r (score)	15.71	3.54
Sleep quality		
PSQI (score)	4.99	1.77
Sleep diary *		
TST (minutes)	415.38	76.18
SOL (minutes)	19.46	27.75
SE (%)	92.02	11.31
Actigraphy *		
TST (minutes)	398.84	72.99
SOL (minutes)	10.74	17.14
SE (%)	85.16	9.52
Thought control strategies		
TCQI-r, Agg Supp (score)	14.66	3.86
TCQI-r, Cogn Dist (score)	12.32	2.98
TCQI-r, Behav Dist (score)	10.11	3.82
TCQI-r, Reapp (score)	15.01	4.13
TCQI-r, Worry (score)	11.39	2.86

Note. MEQ-r = Circadian preference; PSQI = Pittsburgh Sleep Quality Index; TST = total sleep time; SOL = sleep onset latency; SE = sleep efficiency; TCQI-R, Agg Supp = Aggressive suppression; TCQI-R, Cogn Dist = Cognitive distraction; TCQI-R, Reapp = Reappraisal; TCQI-R, Behav Dist = Behavioral distraction; TCQI-R, Worry = Worry. * Data averaged across the week.

## Data Availability

Data supporting the reported results can be obtained by contacting E.S. (enrico.sella@unipd.it).

## Supplementary Materials

The following supporting information can be downloaded at: https://www.mdpi.com/article/10.3390/brainsci13020271/s1, Table S1: Results of the linear mixed-effects models for subjective sleep parameters (sleep diary); Table S2: Results of the linear mixed-effects models for actigraphic sleep parameters; Table S3: Model comparison results. The most parsimonious, best-fit models with the lowest AIC and highest weights were selected appear in bold for each subjective sleep parameter (Sleep diary); Table S4: Model comparison results. The most parsimonious, best-fit models with the lowest AIC and highest weights were selected appear in bold for each actigraphic sleep parameter; Table S5: Pearson’s Correlations between subjective and objective sleep parameters.
