# Supplementary material for: Do Sleep-Related Metacognitive Strategies Shape My Sleep? The Relationships between Strategies for Controlling Sleep-Related Intrusive Thoughts and Subjective and Objective Sleep Quality in Young Adulthood and Older Age
Source: Brain Sci. 2023 Feb 6;13(2):271. doi: 10.3390/brainsci13020271 (PMC9953834; doi:10.3390/brainsci13020271)
Supplement: Supplementary file 1 [file brainsci-13-00271-s001.zip › brainsci-2151296-supplementary.pdf]

SUPPLEMENTARY MATERIAL

Do Sleep-Related Metacognitive Strategies Shape My Sleep? The Relationships between Strategies for Controlling Sleep-Related Intrusive Thoughts and Subjective and Objective Sleep Quality in Young Adulthood and Older Age

DETAILS OF RESULTS – ADDITIONAL TABLES

Table S1. *Results of the linear mixed-effects models for subjective sleep parameters (sleep diary)*

Measure of interest	*Predictors*	*B*	*CI*	*p*
TST	**Intercept**	459.40	402.73 – 516.06	**<0.001**
	**Age**	-0.84	-1.26 – -0.41	**<0.001**
	Sex	7.03	-8.14 – 22.20	0.364
	MEQ-r	1.55	-0.78 – 3.88	0.193
	**PSQI**	**-6.12**	**-10.69 – -1.54**	**0.009**
	**TCQI-r, Agg Supp**	**2.88**	**0.45 – 5.30**	**0.020**
	TCQI-r, Cogn Dist	-1.70	-4.52 – 1.11	0.236
	TCQI-r, Behav Dist	1.35	-1.02 – 3.73	0.263
	TCQI-r, Reapp	0.54	-1.75 – 2.83	0.646
	**TCQI-r, Worry**	-4.13	-6.66 – -1.59	**0.001**
SOL	Intercept	-15.51	-38.06 – 7.05	0.178
	**Age**	0.36	0.19 – 0.53	**<0.001**
	Sex	-4.06	-10.10 – 1.98	0.187
	MEQ-r	0.12	-0.81 – 1.05	0.798
	**PSQI**	**2.48**	**0.66 – 4.30**	**0.008**
	TCQI-r, Agg Supp	0.32	-0.65 – 1.28	0.517
	TCQI-r, Cogn Dist	-0.03	-1.15 – 1.09	0.959
	TCQI-r, Behav Dist	-0.29	-1.23 – 0.66	0.553
	TCQI-r, Reapp	-0.85	-1.76 – 0.07	0.069
	**TCQI-r, Worry**	**1.56**	**0.56 – 2.57**	**0.002**
SE	**Intercept**	99.56	92.71 – 106.42	**<0.001**

	**Age**	**-0.13**	**-0.19 – -0.07**	**<0.001**
	Sex	1.63	-0.67 – 3.94	0.165
	MEQ-r	0.30	-0.06 – 0.65	0.101
	**PSQI**	**-1.48**	**-2.17 – -0.78**	**<0.001**

Note. MEQ-r= Circadian preference; PSQI= Pittsburgh Sleep Quality Index; TST= total sleep time; SOL= sleep onset latency; SE= sleep efficiency; TCQI-R, Agg Supp=Aggressive suppression; TCQI-R, Cogn Dist= Cognitive distraction; TCQI-R, Reapp= Reappraisal; TCQI-R, Behav Dist= Behavioral distraction; TCQI-R, Worry= Worry; TST= total sleep time; SOL= sleep onset latency; SE= sleep efficiency.

Table S2. *Results of the linear mixed-effects models for actigraphic sleep parameters*

Measure of interest	***Predictors***	***B***	***CI***	***p***
TST	**Intercept**	411.73	353.65 – 469.81	**<0.001**
	Age	-0.19	-0.63 – 0.24	0.387
	Sex	10.03	-5.53 – 25.58	0.207
	MEQ-r	1.18	-1.22 – 3.57	0.336
	PSQI	-2.33	-7.02 – 2.36	0.331
	TCQI-r, Agg Supp	-0.61	-3.10 – 1.88	0.631
	TCQI-r, Cogn Dist	-0.66	-3.56 – 2.23	0.654
	TCQI-r, Behav Dist	1.45	-0.98 – 3.89	0.243
	TCQI-r, Reapp	0.35	-2.00 – 2.71	0.768
	TCQI-r, Worry	-1.75	-4.35 – 0.84	0.185
SOL	**Intercept**	4.82	-6.63 – 16.27	0.409
	**Age**	0.07	-0.03 – 0.17	0.162
	**Sex**	**-4.22**	**-8.08 – -0.37**	**0.032**
	MEQ-r	-0.06	-0.65 – 0.53	0.837

	**PSQI**	**1.26**	**0.10 – 2.42**	**0.033**
SE	**Intercept**	110.73	85.34 – 136.11	**<0.001**
	Age	0.10	-0.09 – 0.30	0.298
	Sex	3.08	-3.56 – 9.72	0.363
	**MEQ-r**	**-1.23**	**-2.11 – -0.35**	**0.006**
	PSQI	-0.34	-2.33 – 1.64	0.735
	TCQI-r, Agg Supp	0.51	-0.97 – 1.98	0.498
	TCQI-r, Cogn Dist	-0.05	-1.25 – 1.16	0.940
	TCQI-r, Behav Dist	0.79	-0.67 – 2.25	0.286
	TCQI-r, Reapp	-1.00	-2.26 – 0.26	0.119
	TCQI-r, Worry	-0.89	-2.16 – 0.39	0.173

Note. MEQ-r= Circadian preference; PSQI= Pittsburgh Sleep Quality Index; TST= total sleep time; SOL= sleep onset latency; SE= sleep efficiency; TCQI-R, Agg Supp=Aggressive suppression; TCQI-R, Cogn Dist= Cognitive distraction; TCQI-R, Reapp= Reappraisal; TCQI-R, Behav Dist= Behavioral distraction; TCQI-R, Worry= Worry; TST= total sleep time; SOL= sleep onset latency; SE= sleep efficiency.

Table S3. *Model comparison results. The most parsimonious, best-fit models with the lowest AIC and highest weights were selected appear in bold for each subjective sleep parameters (Sleep diary).*

Measure of interest	***Predictors***	***AIC***	***AIC_w***	***Conditional R^2***
TST	m0: intercept	11570.27	0.00	0.373
	m1: + age, sex, circadian preferences (MEQ-r), self-reported sleep quality (PSQI)	11535.55	0.01	0.378
	**m2: + thought control strategies**	**11528.12**	**0.99**	0.385
SOL	m0: intercept	9475.827	0.00	0.441

	m1: + age, sex, circadian preferences (MEQ-r), self-reported sleep quality (PSQI)	9437.645	0.16	0.446
	**m2: + thought control strategies**	**9434.303**	**0.84**	0.478
SE	m0: intercept	7575.480	0.00	0.436
	**m1: + age, sex, circadian preferences (MEQ-r), self-reported sleep quality (PSQI)**	**7536.554**	**0.99**	**0.441**
	m2: + thought control strategies	7546.161	0.01	0.448

Note. MEQ-r= Circadian preference; PSQI= Pittsburgh Sleep Quality Index; TST= total sleep time; SOL= sleep onset latency; SE= sleep efficiency; TCQI-R, Agg Supp=Aggressive suppression; TCQI-R, Cogn Dist= Cognitive distraction; TCQI-R, Reapp= Reappraisal; TCQI-R, Behav Dist= Behavioral distraction; TCQI-R, Worry= Worry; TST= total sleep time; SOL= sleep onset latency; SE= sleep efficiency.

Table S4. *Model comparison results. The most parsimonious, best-fit models with the lowest AIC and highest weights were selected appear in bold for each actigraphic sleep parameter.*

Measure of interest	***Predictors***	***AIC***	***AIC_w***	***Conditional R^2***
TST	m0: intercept	11555.61	0.01	0.313
	m1: + age, sex, circadian preferences (MEQ-r), self-reported sleep quality (PSQI)	11547.63	0.07	0.320
	**m2: + thought control strategies**	**11542.54**	**0.93**	**0.329**
SOL	m0: intercept	8497.133	0.035	0.404
	**m1: + age, sex, circadian preferences (MEQ-r), self-reported sleep quality**	**8490.509**	**0.951**	**0.410**

	**(PSQI)**			
	m2: + thought control strategies	8498.904	0.014	0.419
SE	m0: intercept	748.5178	0.015	0.786
	m1: + age, sex, circadian preferences (MEQ-r), self-reported sleep quality (PSQI)	742.3785	0.32	0.799
	**m2: + thought control strategies**	**740.9372**	**0.66**	**0.804**

Note. MEQ-r= Circadian preference; PSQI= Pittsburgh Sleep Quality Index; TST= total sleep time; SOL= sleep onset latency; SE= sleep efficiency; TCQI-R, Agg Supp=Aggressive suppression; TCQI-R, Cogn Dist= Cognitive distraction; TCQI-R, Reapp= Reappraisal; TCQI-R, Behav Dist= Behavioral distraction; TCQI-R, Worry= Worry; TST= total sleep time; SOL= sleep onset latency; SE= sleep efficiency.

Table S5. *Pearson's Correlations between subjective and objective sleep parameters.*

	1	2	3	4	5
Sleep diary					
TST (1)	—				
SOL (2)	-0.525^{***}	—			
SE (3)	0.389^{***}	-0.415^{***}	—		
Actigraphy					
TST (4)	0.302^{***}	0.031	0.155	—	
SOL (5)	-0.072	0.191^{*}	-0.094	-0.143	—
SE (6)	0.136	-0.088	0.006	0.656^{***}	-0.375^{***}

Note. PSQI= Pittsburgh Sleep Quality Index; TST= total sleep time; SOL= sleep onset latency; SE= sleep efficiency. $^{*}p < .05$, $^{**}p < .01$, $^{***}p < 001$.
